# LTDNA Evidence on Trial

**DOI:** 10.3389/fgene.2016.00180

**Published:** 2016-10-25

**Authors:** Paul Roberts

**Affiliations:** ^1^School of Law, University of NottinghamNottingham, UK; ^2^Collaborative Innovation Center of Judicial Civilization, Institute of Evidence Law and Forensic Science, China University of Political Science and LawBeijing, China; ^3^Faculty of Law, University of New South WalesSydney, NSW, Australia

**Keywords:** expert evidence, criminal adjudication, LTDNA profiling evidence, comparative criminal procedure, Law-Science, interdisciplinarity

## Abstract

Adopting the interpretative/hermeneutical method typical of much legal scholarship, this article considers two sets of issues pertaining to LTDNA profiles as evidence in criminal proceedings. The section titled Expert Evidence as Forensic Epistemic Warrant addresses some rather large questions about the epistemic status and probative value of expert testimony in general. It sketches a theoretical model of expert evidence, highlighting five essential criteria: (1) expert competence; (2) disciplinary domain; (3) methodological validity; (4) materiality; and (5) legal admissibility. This generic model of expert authority, highlighting law's fundamentally normative character, applies to all modern forms of criminal adjudication, across Europe and farther afield. The section titled LTDNA Evidence in UK Criminal Trials then examines English and Northern Irish courts' attempts to get to grips with LTDNA evidence in recent cases. Better appreciating the ways in which UK courts have addressed the challenges of LTDNA evidence may offer some insights into parallel developments in other legal systems. Appellate court rulings follow a predictable judicial logic, which might usefully be studied and reflected upon by any forensic scientist or statistician seeking to operate effectively in criminal proceedings. Whilst each legal jurisdiction has its own unique blend of jurisprudence, institutions, cultures and historical traditions, there is considerable scope for comparative analysis and cross-jurisdictional borrowing and instruction. In the spirit of promoting more nuanced and sophisticated international interdisciplinary dialogue, this article examines UK judicial approaches to LTDNA evidence and begins to elucidate their underlying institutional logic. Legal argument and broader policy debates are not confined to considerations of scientific validity, contamination risks and evidential integrity, or associated judgments of legal admissibility or exclusion. They also crucially concern the manner in which LTDNA profiling results are presented and explained to factfinders in criminal trials.

## Introduction: an interdisciplinary topic of conversation

As originally conceived, this Research Topic focused on “the interface between forensic scientists and statisticians when calculating likelihood ratios for low template and complex DNA results”^1^. The problem of calculating and interpreting likelihood ratios was thereby implicitly characterized as a bilateral conversation between statisticians and forensic scientists. Of course, on further reflection, there is self-evidently a third major conversation partner in this discussion, namely courts and legal professionals. We might add that criminal “courts” in jurisdictions such as England and Wales comprise a mixture of professional judges and lay fact-finders, though jurists and jurors alike are laypeople when it comes to DNA profiling science. Our Topic Editors observed that “[t]here is a danger for courts if [likelihood ratios] are produced by a black box where the reporting forensic scientist has little input into and less understanding” of statistical methods. The clear (and unsurprising) presupposition is that difficulties associated with Low Template DNA (LTDNA) evidence cannot simply be conceptualized as “technical” questions to be resolved between specialists according to mutually satisfactory methodological criteria. There remains the challenge of communicating the meaning and significance of technical fixes to lay audiences in criminal adjudication. This communicative dimension is a general feature of expert testimony, whether or not it concerns anything properly categorized as “science”^2^.

This article considers two sets of issues pertaining to LTDNA profiles as evidence in criminal proceedings, which are pertinent to all modern legal systems in which this type of evidence is currently or might in future be adduced. The section titled Expert Evidence as Forensic Epistemic Warrant addresses some rather large questions about the epistemic status and probative value of expert testimony in general. The following section on LTDNA Evidence in UK Criminal Trials then examines English courts' attempts to get to grips with LTDNA evidence in recent cases. These efforts might or might not appear impressive to outsiders, but they do, generally speaking, follow a predictable judicial logic—a logic which might usefully be studied and reflected upon by any forensic scientist or statistician seeking to operate effectively in this system of justice. In the spirit of promoting more nuanced and sophisticated interdisciplinary dialogue this article examines judicial approaches to LTDNA evidence and begins to elucidate their underlying institutional logic.

The following discussion adopts the interpretative or hermeneutical method typical of much legal scholarship. It engages with primary and secondary institutional materials—prominently featuring reported criminal appeals in England and Wales—in an attempt both to understand judicial practice and to contribute to the best normative (re)interpretation of legal doctrine and institutions. It might fairly be conceptualized, in methodological terms, as an elucidation of the internal logics of legal argumentation and judicial reasoning, which are often opaque to non-lawyers, even to those such as forensic scientists who regularly participate in criminal investigations and are no strangers to courtrooms. In striking contrast to scientific knowledge, law is delimited by jurisdiction. The second half of this article discusses appellate decisions drawn from two specific, common law jurisdictions: Northern Ireland (applying Northern Ireland law), and England and Wales (applying English law). Whilst each legal jurisdiction has its own unique blend of jurisprudence, institutions, cultures, and historical traditions, there is considerable scope for comparative analysis and cross-jurisdictional borrowing and instruction, the more so in a shrinking world characterized by globalization and cosmopolitan legality. Better appreciating the ways in which UK courts have addressed the challenges of LTDNA evidence may offer some insights into parallel developments in other legal systems. Moreover, the practical challenges posed by forensic science and other expert witness testimony cut across conventional Comparative Law distinctions between “adversarial” and “inquisitorial” procedures or “common law” and “civilian” legal systems. In setting the scene for more detailed doctrinal analysis, the first part of this article presents a generic model of expert authority highlighting law's fundamentally normative mission which applies to all modern forms of criminal adjudication, across Europe and farther afield.

## Expert evidence as forensic epistemic warrant

Criminal trials are practical exercises in reasoning under uncertainty. We want to know what happened; but material facts are contested (otherwise the accused would have pleaded guilty in common law systems). Relevant evidence rationally authorizes or “warrants” particular inferential conclusions. The more probative value evidence has, the more warrant it provides for the conclusion. In traditional common law thinking, the best evidence is the oral testimony of a percipient witness, given on oath, and tested through cross-examination. This category of evidence is regarded as providing the best epistemic warrant for the inferential conclusions supported by the witness's testimony. This doesn't mean to say, of course, that every witness in court is truthful, accurate and reliable. We know, for example, that there can be many kinds of difficulty with eyewitness testimony^3^. But it does not follow, as a general proposition, that we should therefore prefer the testimony of those who did *not* see the incident to testimony from witnesses who did. “Best” does not mean infallible. Evidence adduced in criminal trials is often contested or contradictory, and the factfinder must make the best of it, resolving any enduring doubts in accordance with the applicable burden and standard of proof. In criminal litigation, most (not all) doubts are resolved in favor of the accused, in accordance with the presumption of innocence^4^.

Expert evidence supplies inferential warrant through the argument from authority. The expert says to the court, *trust me, I'm an expert*. The authority paradigm underpinning the inferential logic of forensic expert testimony has five major components: (1) the expert is a genuine expert (competence); (2) in a field in which expertise can be obtained (domain); (3) and has correctly and conscientiously applied authentic domain-specific protocols to produce proffered evidence (methodological validity); (4) in relation to a legally relevant issue (materiality); (5) and in a form that is likely to provide legitimate epistemic warrant for legal adjudication (admissibility). The authority paradigm is generic. It applies to “sciences” as conventionally understood (and in idiomatic English, this generally means “the hard sciences” like physics and chemistry), but also to historical, social and psychological facts, and even to moral and theological reasoning. This 5-fold taxonomy, albeit a necessarily simplifying model, offers a powerful heuristic for teasing out theoretical complexities and practical challenges entailed by the familiar-sounding notion of forensic expertise^5^. For example, components (1) to (3)—competence, domain and methodological validity—interact in interesting ways. The authority paradigm obviously breaks down if the testimony is not proffered by a genuine expert; if the so-called expert is an “incompetent” witness in the common lawyer's sense. But sometimes, it is not so much the qualifications and experience of individual experts that are at stake, but the very possibility of domain expertise. The objection to “expert” witch-finders^6^ or ghost hunters is more fundamental (and less *ad hominem*) than any criticism that individual exponents have not taken the appropriate training courses or gained enough job-related experience.

Methodological validity, component (3), embraces a set of important epistemic considerations arising even in relation to genuine experts in well-established disciplinary domains. The authority paradigm breaks down for different reasons when genuine experts succumb to personal or professional biases, fail to implement pertinent methodological protocols correctly, or purport to speak beyond the boundaries of their domain-specific expertise. It may be difficult in the general run of cases for courts to differentiate between genuinely well-credentialed experts, and plausible-sounding charlatans and shysters^7^. This practical challenge is so much greater, however, in relation to the types of failing encompassed by component (3), where genuine experts are over-reaching in one way or another^8^. Forms of expert testimony incorporating multiple specialist domains, including DNA profiling, pose such dilemmas acutely. Plainly, not every opinion or judgment expressed by an expert properly qualifies as *expert opinion*. Judges may be ill-equipped and trial procedure ill-suited to policing experts' disciplinary boundaries effectively.

Components (4) and (5) of the authority paradigm—materiality and admissibility—introduce further major complexities, in terms of managing the interface between expert knowledge and forensic objectives, concerns and values. A vital distinction is that, whereas components (1)–(3) are essentially epistemic matters, criminal adjudication is fundamentally *normative*. The overriding objective of criminal proceedings is doing justice;^9^ and whilst epistemic considerations are vital ingredients in the mix—we want to convict the guilty, and only them, of the right offence(s)—epistemology is not the proof of the pudding. We only want to convict the guilty *in the right way* (“by due process of law”), not any which way—e.g., by vigilante lynch mob or Dirty Harry policing^10^ in violation of the rule of law. Thus, all evidence, including expert witness testimony, must satisfy fundamental criteria of procedural fairness, transparency, exposure to adversarial testing, and compliance with other basic criteria of the right to a fair trial. Notwithstanding their divergent legal histories, idiosyncratic procedural traditions and distinctive institutional cultures, all 47 Council of Europe nations are bound by a common conception of the fundamental requirements of fair criminal trials under Article 6 of the ECHR^11^ (which is entirely separate from parallel or overlapping EU legal frameworks applicable only to the 28 countries of the “smaller Europe”^12^).

It is at this point in the discussion that, in my experience, lawyers and scientists tend to see things differently; and misunderstandings easily arise. Science investigates empirical matters, and produces factual information about the world. It is epistemic to its core and overwhelmingly instrumental in outlook. The policy paradigm is “curing cancer.” A new drug either works (in part), or it does not. It has particular side-effects (in some degree), or it does not. It can be manufactured by a particular process, or it cannot. Likewise, the DNA collected from the crime scene was either deposited by the accused, or by somebody else; the accused lacked capacity to form the required intention at the material time (e.g., because catatonic or sleepwalking), or he did not; and so on. These are all facts about the (empirical) world; they are either true or false; and they invoke or presuppose causal explanations. This is not to say or imply that “science” always provides unequivocal, certain answers to discrete, well-formulated questions. To the contrary, scientific investigation is inherently uncertain (“experimental”), and conclusions are typically framed in probabilistic terms—whether or not employing explicitly quantitative measurements of uncertainty in numbers or words. But the equivocation introduced by resorting to probability is *epistemic* not ontological: it relates to the status of our knowledge and beliefs about facts in the world, not to the facts themselves (setting aside complications arising from quantum physics and sub-atomic particles not pertinent to the present discussion). Judgments of justice are of an entirely different, normative, order. It is not merely doubtful or uncertain whether, say, it would be *just* or *fair* if Drug X cured cancer; or *just* or *fair* if the accused were the donor of crime scene DNA. Such questions are incoherent. They perpetrate a category error, confusing normative standards with empirical facts.

Criminal adjudication comprises a set of institutionalized practices for determining liability and censuring and punishing criminal wrongdoing^13^. This set of practices is normative through and through. It is not just that epistemic considerations are subject to normative side-constraints, as where we exclude relevant evidence procured by torture irrespective of its epistemic credentials^14^. Epistemic objectives are themselves normatively constituted, in the sense that the standard of adequate epistemic warrant is indexed to the institutionalized practices and objectives of criminal adjudication. So what we require is not “adequate warrant” (sufficient grounds) in the abstract, but *adequate warrant for the purposes of determining criminal liability and censuring and punishing criminal wrongdoers*. By reframing the issue in this way, it should become clearer why expert evidence cannot provide its own epistemic warrant for judicial purposes, no matter how highly the evidence scores on components (1)–(3) of the authority paradigm. Expert witnesses do not decide what evidence is “good enough” for the purposes of criminal adjudication. This is the role of the legally, indeed constitutionally, authorized fact-finder. Furthermore, issues of materiality and strategic application in individual cases are determined by *legal* standards and *judicial* decision-makers, not by the disciplinary standards embraced by particular sciences or expert witnesses. This insight goes to the heart of the truism that forensic science serves justice, not the other way around.

The logic of the authority paradigm and the priority of normative over epistemic considerations in criminal adjudication are general features of all modern legal systems. However, the ways in which resulting interfaces are organized, opportunities exploited, and tensions managed vary considerably from one legal system to the next, working with the grain of local procedural traditions, institutional practices and professional cultures. For example, in the common law world lay fact-finding is still regarded as significant (even though professional judges increasingly predominate), whilst lay input in criminal adjudication is diminished or even non-existent in most Continental juristic traditions. The roles, relationships, and distribution of powers between judges, prosecutors and defence lawyers also vary considerably across legal jurisdictions. In legal systems with stronger adversarial leanings, prosecutors and defence lawyers tend to play a more active role in shaping the course of the proceedings, whereas the “inquisitorial” judge is the dominant figure in other procedural models. Criminal procedure is dynamic and constantly evolving (we have seen major shifts toward a philosophy of activist judicial trial management in England and Wales in recent years, for example), and it is always perilous to over-generalize abstract formal models or to extrapolate too confidently from national traditions. It follows that approaches to expert evidence in general, or to particular types of scientific evidence such as LTDNA, which are utilized successfully in one jurisdiction cannot automatically be expected to operate with the same success, or at all, in a different procedural environment structured by alternative normative priorities. This observation holds *irrespective of the epistemic credentials of expert evidence*, encapsulated in authority paradigm components (1)–(3). Normative pluralism and jurisdictional diversity are inherent features of modern legality requiring detailed local knowledge and careful negotiation, not least on the part of expert witnesses operating in multiple jurisdictions. However, these elements of cultural relativity tend to provoke intuitive resistance form scientists accustomed to prioritizing universal (empirical) scientific truth over national ideology. In one sense, skepticism is justified: sacrificing science to ideology *in general*, and regardless of political variety, leads to Lysenkoism, authoritarianism, crop failure and mass starvation. Nonetheless, the subservience of science to normative criteria is an inherent, fully rationalized and legitimate *requirement* of penal justice.

Two kinds of recurrent problems with expert evidence call for practical solutions in all legal systems. The first is the problem of expert disagreement; the second, more fundamental problem concerns a recurrent dynamic between deference and education in reliance on expertise. What should a court do when expert witnesses disagree? An attractive first option would be to find that, on further investigation, there is no genuine disagreement to resolve, e.g., because one of the protagonists is not really an expert after all, or not an expert in the relevant domain, or because the experts have been fed different factual assumptions by their instructing lawyers, and once these discrepancies have been clarified the ostensible disagreement disappears. But this convenient resolution will not always be possible. In cases of genuine, well-informed, unshakeable disagreement between experts, various strategies are available to the court. One approach would be to accept the disagreement as a forensically significant fact in and of itself and resolve the issue in accordance with the burden and standard of proof (usually, but not invariably, giving the accused the benefit of the doubt in a criminal trial)^15^. A second strategy would be to side-step scientific disagreements by invoking individual experts' respective qualifications, experience and/or testimonial credibility as proxies for the reliability of their evidence, e.g., by adopting the working assumption that the professor or consultant is more likely to be correct than a laboratory technician or medical student. A third possibility is for the court to try to resolve the disagreement for itself. Strategies two and three exemplify the education/deference dynamic in factfinders' reliance on expert evidence. Strategy two entails deferring to the most authoritative expert, as judged by the factfinder (with or without the benefit of further judicial directions). The third strategy initially sounds the most attractive, because it comports with the factfinder's overarching responsibility for determining disputed questions of fact. The obvious problem is that, by definition, the factfinder lacks domain-specific expertise. Can the experts, through their courtroom testimony, effectively educate the factfinder to arrive at its own decision? Perhaps some element of “education” is possible, even in the constrained and most unpromising pedagogical environment of the criminal courtroom, but it seems quite implausible that factfinders in criminal trials could be equipped with sufficient knowledge and insight to resolve disputes between genuine experts with long years of study and extensive practical experience under their belts^16^. Worse, fact-finders' lack of domain-relevant expertise also undercuts strategy two, because how is it possible for jurors to assess the comparative merits of experts' disagreements when their own knowledge of the field is tenuous or non-existent? The worry is that, in the absence of rational criteria for making a determination, factfinders will fall back on irrational proxies for robust epistemic warrant, such as placing their faith in the expert with greater testimonial eloquence or the doctor with the most reassuring bedside manner.

Legal systems resolve these pervasive issues of expertise in their own distinctive ways. At the level of sweeping generalization, common lawyers tend to think that “civilians don't try”^17^ and that inquisitorial judges too readily defer to authoritative court-appointed experts^18^. Civilians, for their part, tend to regard common law criminal procedure as irrational in its preferences for adversarial theatre, excessive technicality and lay over expert (including expert judicial) decision-making^19^. These longstanding debates implicate deep-rooted and enduring controversies, which it would not be profitable to dig into here; save to say that there is no reason for thinking that structures and cultures of criminal adjudication must conform to a single uniform pattern (so long as they adhere to fundamental standards of justice), any more than we should expect rigid, monotonous uniformity in national cuisine, manners or language. Recognition of legitimate scope for national cultural diversity, even in procedural fundamentals, is another major respect in which international, interdisciplinary conversations about law and justice differ markedly from international conversations about science and expertise.

## LTDNA evidence in UK criminal trials

Recent attempts by criminal courts in England and Wales and Northern Ireland to get to grips with LTDNA profiling evidence must be interpreted in light of the conceptual, normative and juridical considerations summarized in the previous section. The five principal components of the authority paradigm and the diversity of national criminal procedures within a shared ECHR framework mandating fair trials, in particular, need to be borne in mind as the exposition unfolds. Just as the entirety of western philosophy has evolved in productive antagonism with skeptical doubt, legal recognition of LTDNA profiling was propelled by challenges to its methodology, epistemic status and evidential reliability. The evolution of English criminal jurisprudence on LTDNA profiling may find some resonances with parallel developments in other legal jurisdictions, and possibly inform legal argument and policy debates in criminal trials and appeals elsewhere.

### (a) Legal recognition

Our story begins in 2007 with the judgment of the Northern Ireland Crown Court in *R* v *Hoey*^20^, in which Weir J, sitting without a jury in a “Diplock” trial court^21^, commented on the reliability of what was then generally referred to as Low Copy Number (LCN) DNA profiling evidence. LCN profiling evidence in this case, generated by the Forensic Science Service (FSS) laboratory in Birmingham^22^, purported to link the accused to explosive devices used in a string of terrorist bombings across Northern Ireland, including “the infamous car bomb explosion that destroyed much of the shopping Centre of Omagh on the afternoon of Saturday, 15 August 1998 with…appalling consequence[s]…leaving permanent and widespread physical and psychological scars.”^23^ During the course of the trial, serious concerns were identified regarding the integrity of the evidential samples collected from crime scenes, which had not initially been taken, handled or stored with DNA profiling in mind. The Court found that “the arrangements within the police in 1998 and 1999 for the recording and storage of items were thoroughly disorganized”^24^ and that “thoughtless and slapdash” exhibit handling and anti-contamination practices extended to the laboratories and staff of Forensic Service Northern Ireland (FSNI)^25^. Mr. Justice Weir considered it “extraordinary” that “knowing that these items had not been collected or preserved using methods designed to ensure the high degree of integrity needed not merely for DNA examination but for the more exacting requirements of LCN DNA, examinations were performed at Birmingham with a view to using them for evidential rather than solely intelligence gathering purposes.” Yet analytical results from DNA profiling had then been “put forward and stoutly defended” at trial “as evidence that the Court might safely rely upon as tending to establish the guilt of the accused.”^26^ As a matter of legal logic, Weir J's decisive conclusion flowed almost ineluctably from the prosecution's failure to establish the integrity of its evidence:

[O]ne police and SOCO witness after another and also Dr. Griffin [of FSNI] had candidly made clear that possible examination for DNA was not in their minds at all as they were collecting, storing, transmitting and dealing with these items in 1998. Why therefore would they then have had present to their minds and been complying with the exacting integrity requirements which reliable DNA examination and most especially that in its LCN form demands? All this [FSNI] must have known very well when it co-operated in searching for and collecting items for LCN examination in Birmingham and again later when the idea of using the results of those examinations as evidence in this trial must have been under discussion. By that stage the problems inherent in the need to prove integrity had plainly come to be appreciated by one or more police officers concerned in this investigation as was shown by the mendacious attempts to retrospectively alter…evidence so as to falsely make it appear that appropriate DNA protective precautions had been taken at that scene.…[H]aving carefully reviewed all the evidence on this issue, I am not in the least satisfied in relation to any one of the items upon which reliance is sought to be placed for the results of their LCN DNA examinations that the integrity of any of those items prior to its examination for that purpose has been established by the evidence. Accordingly I find that that DNA evidence…cannot satisfy me either beyond a reasonable doubt or to any other acceptable standard^27^.

That is to say, in terms of the conceptual framework sketched in the previous section, the DNA profiling evidence lacked adequate epistemic warrant for grounding a criminal conviction, owing to the well-established fact that forensic samples were compromised—at least in the sense that their integrity could not be demonstrably assured.

Weir J's judgment might have stopped there, but instead briefly addressed the validity and merits of LCN profiling techniques themselves, since these had been extensively canvassed during the trial and conflicting expert views had been expressed. Weir J was “concerned at the wide variance in expert opinions, not only as between the Prosecution and Defence but also between the two experts called for the Prosecution,”^28^ as well as by the “manner and content of the response” of the main FSS expert to defence criticisms. This witness appeared to Weir J to be “inappropriately combative as an expert witness and his unwillingness to debate constructively the various matters put to him was unhelpful in the extreme.”^29^ Notice that, in the absence of domain expertise, Weir J predictably falls back on general proxies for testimonial reliability, such as the (not unreasonable) working assumptions that a conscientious and objective scientist will display an open mind and be prepared to debate criticisms and objections in a fair-minded way. A second prosecution expert, by contrast, came over to the Court as “willing to carefully consider the propositions put to him” by defence counsel, such that “his evidence greatly helped to inform and bring some objectivity to the debate.”^30^ Weir J registered “concern about the present state of the validation of the science and methodology associated with LCN DNA and, in consequence, its reliability as an evidential tool” and expressed himself “not satisfied that the publishing of two journal articles describing a process invented by the authors can be regarded without more as having “validated” that process for the purpose of its being confidently used for evidential purposes.”^31^ These remarks were left hanging within the context of an unresolved broader discussion about enhancing procedural frameworks for regulating the admission and uses of scientific evidence in criminal trials. Weir J suggested that “the evidence given in this case by the FSS witnesses reinforces in the clearest way possible the need for urgent attention to this task.”^32^

Being a decision at first instance, *R* v *Hoey* did not create a binding legal precedent (not even in Northern Ireland), and Weir J's remarks on LCN DNA profiling were strictly *obiter dicta*, i.e., not part of the formal legal holding in the case. It was nonetheless a widely reported judgment in a very high profile trial, which sparked much agitated discussion amongst forensic scientists and led the Association of Chief Police Officers (ACPO), in consultation with the Crown Prosecution Service (CPS), to recommend temporary suspension of LCN profiling techniques in criminal investigations and prosecutions pending further inquiry and review. The science of LTDNA analysis, defined as “[a]n ultra-sensitive technique that has the potential to yield a DNA profile from sub-optimal biological samples e.g., Low Copy Number DNA analysis,”^33^ was subsequently examined by an expert panel established by the Forensic Science Regulator and chaired by Professor Brian Caddy. The Caddy Review concluded that “the science supporting the delivery of Low Template DNA (LTDNA) analysis is sound and that the three companies…providing this service to the Criminal Justice System have validated their processes in accord[ance] with accepted scientific principles.”^34^ But it was noted that “regardless of which signal enhancement method is selected, the problems of allele drop out due to stochastic effects in the presence of low quantities of template and that of increase[d] noise will occur in sub-optimal DNA samples,”^35^ and the concerns expressed by Weir J in *Hoey* regarding the absence of reliable validation were characterized as “well-founded.”^36^ Furthermore:

Interpretation of the results is complex for two reasons: the statistics are challenging and probably hard to comprehend by a non-specialist and the decision how and when to apply certain statistical methods has not yet reached a clear consensus…the challenges in terms of statistical interpretation of the data and in communicating them to a largely innumerate criminal justice system should not be under-estimated, nor should the importance of earning and maintaining public confidence in the system^37^.

These observations appropriately acknowledge the broader institutional context and social expectations of evidential (epistemic) warrant for criminal verdicts. The Caddy Review recommended that “any LTDNA profile should always be reported to the jury with the caveats: that the nature of the original starting material is unknown; that the time at which the DNA was transferred cannot be inferred; and that the opportunity for secondary transfer is increased in comparison to standard DNA profiling.”^38^ Crucially, Caddy expressed the opinion that matches for LCN DNA profiling should be reported at the sub-source, genetic level only. Consequently, it would be “inappropriate to comment upon the cellular material from which the DNA arose or the activity by which the DNA was transferred.”^39^

A follow-up report issued by the Forensic Science Regulator concurred that “the science underpinning the LTDNA analytical services, as provided to the CJS [criminal justice system], is sound and that…suppliers offering such services have properly validated their processes. There is no flaw inherent in the process which prevents its use within the CJS.”^40^ Although there remained “key areas where improvements can be made…probably most importantly, the interpretation of the evidence,”^41^ the Regulator stressed that scope for improvement “does not mean that the approach should not be employed within the CJS”:

As long as the scientist reporting the results of LTDNA analysis complies with the duties and obligations placed on expert witnesses the CJS will appreciate the nature and value of the evidence provided^42^.

This was the state of play, in technical and policy circles, when the Court of Appeal in England and Wales came to consider the status of LTDNA evidence in criminal trials in a clutch of criminal appeals in 2009 and 2010, beginning with *R* v *Reed*^43^.

### (b) Authoritative rulings

The case popularly known as *Reed and Reed* actually comprised two conjoined appeals arising from separate trials, both of which involved challenges to LCN DNA profiling evidence. In *Reed* itself, genetic material was recovered from pieces of plastic which the prosecution contended had broken off from a knife handle employed as the murder weapon. In *Garmson*, the accused was identified as the rapist from small amounts of DNA deposited on the victim's lips, undergarments and tampon. Primed by Weir J's widely reported reservations in *Hoey* and the Caddy Review's findings, the Court of Appeal—led by Thomas LJ, who has since been promoted to Lord Chief Justice—embarked upon a thorough reconsideration of LCN and LTDNA profiling evidence, and directed the parties to assist the Court, within the framework of judicially managed pre-trial case preparation implemented by the Criminal Procedure Rules (CrimPR) since 2005 (and regularly updated). These exchanges produced the following fixed points of agreement: (1) the “standard kit” employed in DNA profiling using the SGM+ system was “designed optimally to produce a full profile on 1 ng which is the approximate equivalent of 160 human somatic cells which typically can be visualized in a tiny blood spot”^44^; (2) “[P]articularly where no identifiable body fluid is present, the amount of DNA present may be as low as the equivalent of that contained in one body cell. Where a sample is measured to be less than what is required to generate a profile using the standard SGM+ test, then Low Template DNA analysis is often undertaken;”^45^ (3) “[T]he stochastic effects may be such that no reliable profile can be generated. The FSS had found that in a very high proportion of profiles obtained using the LCN process the profiles were not capable of robust and reliable interpretation because of stochastic variations.”^46^

Drawing on the technical data and expert opinions canvassed before it and adduced in evidence, the Court of Appeal noted the importance of “the stochastic threshold” at which “the profile is unlikely to suffer from stochastic effects (such as allelic drop out…) which prevent proper interpretation of the alleles.”^47^ Below the stochastic threshold, it is, at the very least, debateable whether analytical results can support meaningful findings, owing to the “noise” generated by uncontrolled random effects. The question then becomes, how much DNA is required to meet this stochastic threshold? The Court of Appeal had heard differing expert views, but prevailing opinion (“in the absence of new scientific evidence”) placed it within the range 100–200 picograms^48^. In the light of this (albeit, possibly temporary and unstable) scientific consensus, the Court of Appeal in *Reed* announced the following principles of admissibility:

[A] challenge to the validity of the method of analysing Low Template DNA by the LCN process should no longer be permitted at trials where the quantity of DNA analysed is above the stochastic threshold of 100–200 picograms.…There may be cases where reliance is placed on a profile obtained where the quantity of DNA analysed is within the range of 100–200 picograms where there is disagreement on the stochastic threshold on the present state of the science. We would anticipate that such cases would be rare and that, in any event, the scientific disagreement will be resolved as the science of DNA profiling develops. If such a case arises, expert evidence must be given as to whether in the particular case, a reliable interpretation can be made. We would anticipate that such evidence would be given by persons who are expert in the science of DNA and supported by the latest research on the subject. We would not anticipate there being any attack on the good faith of those who sought to adduce such evidence^49^.

Here we see the Court of Appeal (literally) laying down the law in relation to the admissibility of LTDNA profiling evidence. There is no general statutory test governing the admissibility of expert evidence in England and Wales. Admissibility is governed by common law principles,^50^ which the courts are both entitled and duty-bound to develop^51^. Strictly speaking, the Court's remarks about evidence under the stochastic threshold are *obiter*, because the DNA evidence in both appeals in *Reed* was above the threshold, and the appeals were ultimately argued and determined on issues of transference and persistence of DNA traces, not on the validity of profiling techniques. However, this (technical) legal objection would predictably fail to gain judicial traction in subsequent cases, given the institutional status and authority of the *Reed* judgment. It would be perfectly evident to experienced lawyers that a senior court was deliberately articulating guidance to be followed in future criminal trials and appeals, with the firm expectation of compliance.

The admissibility principles propounded by the Court of Appeal in *Reed* are interesting at a number of levels. They are animated by the strong desirability of providing clear and reasonably determinate guidance to prosecutors, defence lawyers and trial judges in the conduct of criminal litigation. It would hardly be efficient to try to re-litigate the existence and calibration of stochastic thresholds in each and every criminal trial involving LTDNA profiling evidence, and it would—to say the least—be highly undesirable for individual courts to be improvising their own, quite possibly discrepant, thresholds, depending partly on which expert witnesses happened to testify in particular trials and how their evidence was received and assessed by individual trial judges in admissibility determinations (and hostage to further contingencies, including whether admissibility was, in fact, challenged in the instant case^52^). The problem is that there is no readily available institutional mechanism for establishing “legislative facts,” such as the nature of stochastic thresholds for LTDNA profiling evidence, in English criminal proceedings. This is not the sort of thing that could be included in a Code of Criminal Procedure, even if we had one (which, if one has in mind the standard continental model, we don't). So the Court of Appeal is obliged to step into the void and take responsibility for standard-setting upon itself. However, this is slippery and even perilous territory. Can the law plausibly dictate standards of scientific validity, even for its own juridical purposes? The Court of Appeal is primarily concerned with adjudicating questions of *law*, not fact. There is some flexibility, inasmuch as the Court of Appeal makes classificatory choices as the arbiter of what qualifies, in law, as “questions of fact” and “questions of law.” But scientific facts themselves, as opposed to the legitimacy of their forensic uses, cannot be subjected to the normative dictates of law, on pain of reversion to ideology and Lysenkoism.

The judgment in *Reed* makes extensive reference to the Caddy Review^53^ and the input of the Forensic Science Regulator, reflecting a notable emergent symbiosis. Systematic reviews of scientific issues by expert practitioners and independent regulators will almost certainly form sounder technical conclusions, and supply superior epistemic warrant for legal decision-making on scientific questions, than comparable efforts by appellate courts (even those staffed by relatively knowledgeable and scientifically literate judges), examining the facts of particular cases within the constrained institutional parameters of criminal litigation. Recognizing this, the Court of Appeal in *Reed* lent its judicial authority to conclusions arrived at extra-judicially which, in the absence of the Court's imprimatur, would have been relegated to the marginal jurisprudential status of supporting material for expert witness testimony. In this mutually obliging fashion, the Court of Appeal acquires credibility for its scientific conclusions whilst simultaneously conferring judicial kudos on the Regulator. Yet precisely because we are dealing with *scientific facts*, forensic closure cannot be permanent or complete. What happens if prevailing scientific opinion shifts?^54^ This is hardly a remote possibility in rapidly developing fields such as DNA profiling technology (does anybody still remember “genetic fingerprinting,” southern blotting and autoradiographs?^55^). Mindful of the risk of petrifying the law's approach to stochastic thresholds, the Court of Appeal inserted the rider “in the absence of new scientific evidence” into its admissibility principles. However, this is effectively an invitation for trial counsel to argue that they do, indeed, have new scientific evidence at their disposal; and it would not be entirely surprising if time spent researching the relevant academic journals reaped forensic rewards for enterprising trial lawyers. Conversely, if the opportunity for challenge in the light of new evidence had been closed down, later shifts in scientific understandings of stochastic thresholds would have resulted in appeals against conviction on the basis of “fresh evidence.” It is bred into common lawyers that circumstances alter cases and that each set of facts presents its own unique, and eminently distinguishable, characteristics. The Court of Appeal consequently never says “never” in relation to the scope for challenging prevailing scientific wisdom; a caution entirely vindicated by the fact that some very longstanding practices of forensic science (not to mention transient enthusiasms^56^) have turned out to lack sound methodological foundations, and some (like the old “points” system for declaring fingerprint matches) have lately been abandoned^57^.

The Court of Appeal's further “anticipation” (read: directive) that evidence pertaining to stochastic thresholds “would be given by persons who are expert in the science of DNA and supported by the latest research on the subject” might sound like no more than a reiteration of common sense legal orthodoxy, extrapolating from the competency and domain components of the standard authority paradigm for expertise outlined in the previous section. In fact, these loaded remarks were intended to signal the Court's impatience with defence testimony challenging the validity and inferential logic of DNA profiling evidence, based not on direct practical experience in profiling, but on generic principles of scientific validation, methodology and inferential logic. Of one defence expert witness, the Court of Appeal remarked:

He gives evidence with a degree of gravitas and fluency that is impressive and is able to explain concepts clearly. However, his expertise on the interpretation of DNA profiles is limited, without any relevant first hand laboratory or research experience. He is not qualified to make a scene of crime investigation…Whilst it is impossible to understand how he had sufficient expertise to be able to give evidence in *R* v *Hoey*, let alone to assist in the attack made in that case on the LCN process, he has given evidence in so many Low Template DNA cases since then on the strength of the observations in *R* v *Hoey* that he has acquired a degree of experience from these cases, his discussion with others and his reading of papers. We retain clear reservations about the extent of his expertise in relation to DNA profiles…^58^

In relation to a second defence expert, the Court complained that “his experience is of a different jurisdiction where the scientist who gives evidence may have a narrower type of expertise and the scope of evidence an expert can give may not be the same as the scope in this jurisdiction.…[H]is experience was not based on the work of a forensic scientist in this jurisdiction who attends both the scene of the crime and supervises the laboratory work.”^59^ These attempts to prioritize hands-on forensic experience over academic research and theorizing are not entirely convincing. Given that expertise is domain-specific, careful attention needs to be given to the grounds, parameters and content of any particular expert's evidence. Courts should certainly be chary of receiving “expert” testimony about laboratory conduct and protocols from somebody who has never worked in a laboratory. By parity of reasoning, however, practitioners are not necessarily good authorities on questions of policy or theory. An example highly pertinent to the present discussion is that a geneticist could be highly accomplished and very experienced in DNA profiling techniques without necessarily having acquired a firm grasp of the statistical foundations and probabilistic methods employed in assessing likelihood ratios for complex mixtures or partial profiles (or even, for that matter, in generating random match probabilities for straightforward single profiles)^60^. Another background consideration possibly at work here is English courts' intuitive suspicion of expert testimony attempting to instruct fact-finders in relation to general considerations of logic and inferential reasoning. Such testimony is often viewed, not without justification, as potentially trenching on the jury's constitutional prerogatives in fact-finding^61^. But the practitioner/theoretician continuum is orthogonal to that concern; and expert competence must always be assessed relative to domain and materiality. The danger is that in ostentatiously rejecting one false proxy for testimonial reliability (a witness's gravitas and fluency), the Court of Appeal in *Reed* may have allowed itself to be gulled by another (“local practitioners always know best”).

### (c) After *Reed*

Lord Justice Thomas' prediction, or pious hope, that cases involving LTDNA profiles arguably under the stochastic threshold “would be rare” was soon put to the test. In *R* v *Broughton*^62^ an animal rights activist was convicted of planting incendiary devices in buildings owned by two Oxford colleges. It was common ground that the attacks were a protest against animal experiments by university scientists. A central plank of the prosecution's (entirely circumstantial) case against Broughton was an LTDNA profile derived from match stalks which had formed part of the fuse mechanism of improvised incendiary devices (bottles filled with petrol) used in one attack. The amount of genetic material recovered from the crime scene was < 100 picograms. This was insufficient to generate any usable results from standard profiling techniques. However, by running multiple enhanced LTDNA analyses and combining their results to produce a “cleaned up” profile, a forensic scientist was able to identify 20 alleles shared in common with the accused. This produced a random match probability, comparable to RMPs for standard profiling, of < 1 in 1 billion.

One argument advanced on appeal was that *Reed* had already decided that profiles below the stochastic threshold range of 100–200 picograms are inadmissible in English law. The Court of Appeal in *Broughton* made short work of the faulty logic in this submission:

The appellant's submission is…founded upon a misunderstanding of the decision in *Reed & Reed*. This court recognised that in the current state of technology there is a stochastic threshold between 100 and 200 picograms above which LTDNA techniques…can be used to obtain profiles capable of reliable interpretation. Specifically, the court observed that above this threshold a challenge to the validity of the method of analysing LTDNA by the LCN process should not be permitted in the absence of new scientific evidence. However, the court did not hold or make any observation to the effect that below the stochastic threshold DNA evidence is *not* admissible^63^.

The defence argument that LTDNA profiles below the stochastic threshold are automatically inadmissible resurfaced in a second appeal heard later in the year, involving DNA mixtures from more than one donor, and it was once more emphatically rejected. The Court of Appeal reiterated that “what mattered was the quality of the minor profiles and not the quantity.…[P]rofiles obtained from < 200 picograms can be reliable. It is reliability that is the issue, not the quantity, though plainly the quantity is relevant…to the consideration of stochastic effects.”^64^

A more promising line of attack was to challenge the profiling evidence on its own scientific merits. The Court of Appeal recognized that the profiles adduced in *Broughton* “were derived from unquantified samples of DNA of < 100 picograms and that this raised entirely legitimate grounds for scientific dispute which the appellant was right in testing before the judge.”^65^ Prevailing scientific understanding was summarized as follows:

[T]here is now a considerable body of opinion from respected independent scientists and the Forensic Science Regulator that LTDNA techniques, including those used to generate the profiles relied upon by the Crown in this case, are well understood, have been properly validated and are accepted to be capable of generating reliable and valuable evidence. At these very low levels of DNA, the dangers presented by the possibility of stochastic effects, including allelic drop-out, drop-in and stutter are very real and must be fully appreciated, but they may often be addressed by repeating the process a number of times…^66^

Observe, again, the instrumental role of the Forensic Science Regulator in authenticating the underpinning science and validation processes. If—and for as long as—the Regulator is satisfied on these technical questions, the courts are likely to follow her^67^ lead. Having noted the *potential* shortcomings of LTDNA profiles, however, the Court of Appeal was satisfied that the pertinent issues had been fully ventilated in the trial and that the evidence actually generated and adduced in the instant case had been properly explained and vindicated by competent experts:

[A]ll of the consensus alleles match those in the appellant's profile. In other words, the consensus profiles do not suggest the procedures suffered from drop-in or stutter such as to render the results inherently unreliable. Indeed, this is reflected in the statistics [sic] derived from the consensus profiles to which we have referred and about which there was no dispute. At their most powerful and when derived from all duplicated components, these give rise to the match probability of < 1 in 1 billion. We believe that these were all matters properly admitted in evidence^68^.

It is important to appreciate that criminal appeals in England and Wales are not re-trials, as they are in many continental European jurisdictions. The Court of Appeal in England and Wales performs an essentially reviewing function, and is primarily concerned with the legality and fairness of trial proceedings, not with the accuracy of their outcomes. Crucially, the Court of Appeal does not second-guess jury verdicts. Once the Court in *Broughton* had satisfied itself that the trial judge had adopted the correct approach to assessing the admissibility of LTDNA evidence, that question was settled for the purposes of this appeal. But the Court still had something it wanted to get off its chest, and the Judgment had a sting in the tail.

The Court signaled a general concern about defence tactics in challenging the credibility and “integrity” of experts presenting DNA profiling evidence:

Whatever may be the position in other jurisdictions, it is the duty of an advocate and an expert in this jurisdiction not to embark upon an attack on the integrity of other experts unless there is an evidential basis for doing so. There was none in this case. The attack made on the integrity of LGC Forensics and Cellmark was without foundation and should never have been made…[T]here can well be a difference of opinion between experts on LTDNA, but there should be no question of the good faith of those involved in LTDNA being put in issue. This is a case where there is a proper disagreement between experts but the course taken by those giving evidence on behalf of the appellant went into matters for which there was no foundation. Not only was the attack on the good faith of the Crown's witness wholly deplorable and unwarranted, but it also was a great disservice to the appellant's case^69^.

The Court is here saying that not only are such credibility attacks contrary to ethical standards of advocacy, and therefore liable to get counsel into hot water with their professional regulator^70^, but also likely to back-fire by harming the defendant's prospects in the instant case. The threat is clear, but whether advocates will pay any attention to it, less so. In *Broughton*, specifically, “an attack was made…on the integrity of LGC Forensics; it was alleged that their commercial interests and influence over their case workers had tainted their professionalism and objectivity. LGC Forensics were underestimating the problems which were associated with LTDNA and promoting its viability for financial reasons.”^71^ Counsel presumably thought it legitimate to draw the jury's attention to possible conflicts of interest in the production of expert evidence, which is now an embedded structural feature of a marketplace dominated by commercial providers following the demise of the FSS^72^. The Court of Appeal's message is that unfocused and entirely unsubstantiated insinuations of commercial corruption will not be tolerated. The situation would presumably be different if there were material evidence that a particular expert's objectivity or impartiality might have been compromised by commercial incentives. In relation to *judicial* impartiality, the court must be manifestly, not merely actually, unbiased^73^, so that justice is *seen* to be done. How much of this expectation carries over to expert witnesses utilizing techniques from which their employers derive a commercial advantage is a nicely balanced question.

Once evidence has been ruled admissible, attention shifts to its uses and probative value in the trial. An important dimension of evidentiary regulation, and one which has been assuming greater prominence in many common law jurisdictions including England and Wales over the last several decades, concerns judicial directions to the jury^74^. English law contains an expanding corpus of “forensic reasoning rules”^75^ instructing factfinders how they must, may or should not utilize particular types and pieces of evidence, which inferences are rationally available and which are legally forbidden. A number of these rules or guidelines pertain to expert evidence in general^76^, and to DNA evidence in particular^77^. This is where the case against *Broughton* unraveled on appeal.

The trial judge in *Broughton* was faced with the task of directing the jury in relation to a disagreement between the expert witnesses regarding the possibility that the DNA sample in question may have been a mixed profile. The expert witness called by the prosecution testified that she was satisfied, on the basis of her experience, that rogue profiling results obtained during the analytical process could be set aside as artefactual stochastic effects. Expert evidence adduced by the defence challenged this conclusion. It was argued that profiling results were consistent with the presence of an unidentified donor, and since the possibility of a mixed sample could not be ruled out, the match probabilities quoted by the prosecution's expert were invalid. The trial judge in his summing-up reminded the jury of this disagreement, which had been characterized as a legitimate difference of opinion between genuine experts. “In other words,” he explained, “there is no, as it were, answer at the back of the book. There is no independent machine if people hold contrary views to tell you in these circumstances who is right and who is wrong. It is a question of expert evidence and scientific judgment…”^78^ The judge added that, if the jury were not satisfied by the prosecution expert's expression of scientific judgment, then her “statistics”^79^ could not be relied upon, and the jury could not substitute its own calculations “because you are not experts.”^80^ In that event, the jury would need to approach the matter cautiously, assessing the probative value of DNA evidence in the absence of any quantified RMP.

Readers of this scientific journal might well be thinking that this direction was incoherent, as a DNA “match” may be close to meaningless, or at least dangerously misleading, in the absence of a valid RMP. The Court of Appeal thought so, too, and concluded “with considerable regret”^81^ that the appeal must be allowed and Broughton's conviction quashed on this, relatively narrow, ground:

[T]he judge…fell into error in directing the jury that, in those circumstances, they could reach their own conclusions on the DNA evidence. It is fair to say that the judge urged the jury to exercise caution and be very careful in arriving at firm conclusions because they were not experts in statistics. However, we believe that only served to emphasise the void in which they were left. They had no guidance from the experts and no guidance from the court to enable them to conduct an evaluation of the evidence for themselves.…[T]he judge ought to have directed the jury that if [the prosecution's expert] was wrong in her conclusion that the DNA profiles were single rather than mixed, then on the only evidence before the court at the trial the DNA evidence must be disregarded. The judge having failed to do so, the jury may well have embarked upon a task of evaluation for which they were not equipped. This means their verdict cannot be regarded as safe^82^.

*Broughton* underlines the point that admissibility is not the only important evidentiary issue raised by LTDNA profiles. The way in which profiling evidence is communicated to lay factfinders is also of fundamental importance if jury verdicts are to secure adequate epistemic warrant and broader normative legitimacy. In all other respects, the trial judge's “admirable summing up” in *Broughton* had “expertly addressed all the evidence and the complex issues in clear terms about which no complaint…could possibly be made.”^83^ A single slip was fatal. Summing up in relation to relatively novel and somewhat complex technologies like LTDNA profiling evidence is evidently a minefield for trial judges. Without a firm grasp of *both* the underlying science of profiling *and* the statistical foundations and probabilistic logic of valid RMPs, trial judges may inadvertently put a foot wrong, with potentially tragic consequences.

This case history might be interpreted, especially by readers more accustomed to inquisitorial procedures (scientists and civilian jurists alike), as a cautionary tale about the hazards of disaggregated tribunals in criminal adjudication and the perils of fastidiously microscopic appellate scrutiny of the wording of judicial directions to juries. These charges are not without substance; but the common lawyer has this riposte. In the absence of any parallel procedure in continental criminal trial proceedings, wherein lies the assurance that judges have any better understanding of the logical foundations of LTDNA evidence and can competently assess its probative value? Do reasoned judgments typically contain sufficiently detailed “motivations” to enable such assessments to be made, by an impartial observer or by the public at large? One could only begin to answer such questions through sustained research and on a jurisdiction-by-jurisdiction basis, but my own fragmentary and partly anecdotal acquaintance with judicial practice in continental Europe suggests that these are pertinent questions to add to our shared research agenda.

## Conclusion

Forensic DNA profiling demands cooperative interdisciplinary expertise in forensic science, statistics and law. This article has reviewed UK courts' responses to LTDNA profiling, starting with initial skepticism in *R* v *Hoey*,^84^ but—with the benefit of more considered official review and expert input—quickly producing authoritative statements endorsing admissibility. English courts proceeded in accordance with their tried-and-tested pragmatic method of *ad hoc* development of common law tests, approaching LTDNA profiling evidence in much the same way as DNA evidence itself was first addressed 30 years ago^85^. Some loose ends left dangling by the Court of Appeal in *Reed*^86^ were tied up in *Broughton*, to produce the following doctrinal conclusion (if it is possible to create a legal precedent in relation to questions of fact, this is it):

[T]he science of LTDNA is sufficiently well-established to pass the ordinary tests of reliability and relevance and it would be wrong wholly to deprive the justice system of the benefits to be gained from the new techniques and advances which it embodies, in cases where there is clear evidence…that the profiles are sufficiently reliable^87^.

Reliability, moreover, is primarily a function of the *quality* of the profiling evidence in the instant case, as vouchsafed by experienced experts. There is no arbitrary stochastic threshold above which LTDNA evidence is admissible, and below which it is automatically excluded.

There is, however, much more to be gleaned from English jurisprudence on LTDNA profiling evidence than these “headline” *rationes decidendi* (formal legal holdings). Judgments rendered by common law courts are complex pieces of legal literature that must be interpreted against a backdrop of “thick” institutional practice and cultural meaning. Some of the factors in play, including the structural logic of the argument from authority sketched in the first part of this article and the priority of normative over epistemic considerations in criminal adjudication, are universal features of modern legal systems. Other factors reflect more local dynamics pertaining to the structural logic of criminal procedure, national legal traditions, and broader features of culture and society (including those features inflecting local apprehensions of adequate epistemic warrant for criminal verdicts). The second half of the article surveyed the principal arguments and judicial rationales that have been deployed in English criminal appeals concerned with LTDNA profiling evidence, pointing out their broader institutional context and resonances and explaining why some gained traction whilst others were rejected. The issues, we saw, are not confined to considerations of scientific validity, contamination risks and evidential integrity, and associated judgments of legal admissibility or exclusion. They also crucially concern the manner in which LTDNA profiling results are presented and explained to lay factfinders in criminal trials.

If opinions differ concerning the adequacy of English courts' responses to LTDNA evidence, this may in part reflect divergent understandings of the deeper structural logic and values of criminal adjudication. These deeper structures are always engaged, and ought to be elucidated and consciously considered, whenever the admissibility and uses of expert evidence are placed under the policy microscope or raise novel legal issues for courts. Because policy questions are fundamentally normative (within the domain of political morality) rather than factually empirical or “scientific,” legal jurisdictions must, in the final analysis, decide what is best for themselves, within the broad parameters of international legal consensus on fundamental rights and democratic values and in harmony with local juristic traditions and cultures. But just as surely as the fact that technical standards of DNA profiling or statistical science cannot dictate the terms of criminal justice, modern legal systems committed to post-Enlightenment conceptions of fact-finding and proof must necessarily rely on the best available scientific and other technical advice, communicated via competent, domain-specific expert evidence, to underpin the rationality (*qua* epistemic warrant) of criminal adjudication.

## Author contributions

The author confirms being the sole contributor of this work and approved it for publication.

### Conflict of interest statement

The author declares that the research was conducted in the absence of any commercial or financial relationships that could be construed as a potential conflict of interest.

## Footnotes

^1^This was the title of the Research Topic originally announced on the Frontiers website, and in my invitation to contribute to it, and was still the current version when I first submitted this paper (11 March 2016). Now see Sue Pope and Alex Biedermann, “Research Topic: The Dialogue Between Forensic Scientists, Statisticians and Lawyers about Complex Scientific Issues for Court,” Frontiers, http://journal.frontiersin.org/researchtopic/4000/the-dialogue-between-forensic-scientists-statisticians-and-lawyers-about-complex-scientific-issues-f (accessed 17 May 2016).

^2^Generally, see Paul Roberts (ed.), *Expert Evidence and Scientific Proof in Criminal Trials* (Ashgate, 2014).

^3^See e.g., Richard A. Wise, Clifford S. Fishman and Martin A. Safer, “How to Analyze the Accuracy of Eyewitness Testimony in a Criminal Case” (2009) 42 *Connecticut Law Review* 435.

^4^The leading case in English law is (still) *Woolmington* v *DPP* [1935] AC 462, HL. However, the concept of “presumption of innocence” is complex and open to a range of normative and epistemic interpretations. See further, Paul Roberts, “Loss of Innocence in Common Law Presumptions” (2014) 8 *Criminal Law and Philosophy* 317.

^5^Also see Susan Haack, *Evidence Matters: Science, Proof and Truth in the Law* (Cambridge UP, 2014); Harry Collins, *Are We All Scientific Experts Now?* (Polity, 2014); Harry Collins and Robert Evans, *Rethinking Expertise* (Chicago UP, 2007).

^6^For pertinent historical context, see Malcolm Gaskill, “Witchcraft and Evidence in Early Modern England” (2008) 198 *Past and Present* 33; Gregory Durston, *Witchcraft and Witch Trials: A History of English Witchcraft and its Legal Perspectives, 1542 to 1736* (Barry Rose, 2000).

^7^Although in no sense representative, the literature contains examples of truly egregious malpractice in pockets of forensics: see e.g., Paul C. Giannelli, “The Abuse of Scientific Evidence in Criminal Cases: The Need for Independent Crime Laboratories” (1997) 4 *Virginia Journal of Social Policy and the Law* 439.

^8^Cf. *Meadow* v *General Medical Council* [2007] QB 462, [2006] EWCA Civ 1390; Richard Nobles and David Schiff, “Misleading Statistics within Criminal Trials – The Sally Clark Case” (2005) 2(1) *Significance* 17.

^9^This is now an explicit normative requirement in English law: Criminal Procedure Rules 2015, r.1. For broader contextualizing discussion, see Paul Roberts, “Groundwork for a Jurisprudence of Criminal Procedure” in R. A. Duff and Stuart Green (eds.), *Philosophical Foundations of Criminal Law* (OUP, 2011).

^10^For idiomatic applications across the political spectrum, see e.g., Russell Dean Covey, “*Miranda* and the Media: Tracing the Cultural Evolution of a Constitutional Revolution” (2007) 10 *Chapman Law Review* 761; Joëlle Anne Moreno, “What Happens when Dirty Harry Becomes an (Expert) Witness for the Prosecution?” (2004) 79 *Tulane Law Review* 1; Richard Nobles and David Schiff, “Due Process and Dirty Harry Dilemmas: Criminal Appeals and the Human Rights Act” (2001) 64 *Modern Law Review* 911; Michael Stokes Paulsen, “Dirty Harry and the Real Constitution” (1997) 64 *University of Chicago Law Review* 1457; Carl B. Klockars, “The Dirty Harry Problem” (1980) 452 *Annals of the American Academy of Political and Social Science* 33.

^11^For general elucidation, see John D. Jackson and Sarah J. Summers, *The Internationalization of Criminal Evidence* (CUP, 2012); Paul Roberts and Jill Hunter (eds.), *Criminal Evidence and Human Rights* (Hart, 2012).

^12^Cf. Paul Roberts, “From Extradition to Surrender: EU Criminal Law and Comparative Legal Method” (2014) 53 *Howard Journal of Criminal Justice* 545; Jacqueline Hodgson, “EU Criminal Justice: The Challenge of Due Process Rights within a Framework of Mutual Recognition” (2011) 37 *North Carolina Journal of International Law and Commercial Regulation* 308.

^13^Generally, see Paul Roberts (ed.), *Theoretical Foundations of Criminal Trial Procedure* (Ashgate, 2014).

^14^*A* v *Secretary of State for the Home Department (No 2)* [2006] 2 AC 221, [2005] UKHL 71; *Gäfgen* v *Germany* (2011) 52 EHRR 1, ECtHR (GC). For discussion, see Paul Roberts, “Normative Evolution in Evidentiary Exclusion: Coercion, Deception and the Right to a Fair Trial” in Paul Roberts and Jill Hunter (eds.), *Criminal Evidence and Human Rights* (Hart, 2012).

^15^This strategy was suggested in *R* v *Cannings* [2004] 1 WLR 2607, [178] (CA), where Judge LJ advised prosecutors and trial judges that “if the outcome of the trial depends exclusively or almost exclusively on a serious disagreement between distinguished and reputable experts, it will often be unwise, and therefore unsafe, to proceed.” But cf. *R* v *Hookway and Noakes* [2011] EWCA Crim 1989.

^16^These issues are well-debated in the legal literature: see further, Ronald J. Allen, “Expertise and the *Daubert* Decision” (1994) 84 *Journal of Criminal Law and Criminology* 1157; Edward J. Imwinkelreid, “The Next Step in Conceptualizing the Presentation of Expert Evidence as Education: the Case for Didactic Trial Procedures” (1997) 1 *International Journal of Evidence and Proof* 128; Gary Edmond, “The Next Step or Moonwalking? Expert Evidence, the Public Understanding of Science and the Case Against Imwinkelreid's Didactic Trial Procedures” (1998) 2 *International Journal of Evidence and Proof* 13; Imwinkelreid, “Correspondence: Didactic Trial Procedures” (1998) 2 *International Journal of Evidence and Proof* 205.

^17^William Twining, “Civilians Don't Try: A Comment on Mirjan Damaska's “Rational and Irrational Proof Revisited”” (1997) 5 *Cardozo Journal of International and Comparative Law* 69.

^18^MN Howard QC, “The Neutral Expert: A Plausible Threat to Justice” [1991] *Criminal Law Review* 98. Such stereotypes are not without empirical foundation: cf. Chrisje Brants, “Wrongful Convictions and Inquisitorial Process: the Case of the Netherlands” (2012) 80 *University of Cincinnati Law Review* 1069, 1111 (observing that, “[j]udges may be inclined to give too much weight to expert testimony and forensic evidence (especially true of DNA).…[I]t is perhaps more problematic that judges will generally have at their disposal the evidence of only one expert.…[T]he routine absence of an expert for the defence means that the court is dependent upon its own, often amateur, evaluation of the evidence”).

^19^See further, J. R. Spencer, “Court Experts and Expert Witnesses: Have We a Lesson to Learn from the French?” (1992) 45 *Current Legal Problems* 213; William T. Pizzi, *Trials Without Truth* (NYU Press, 1999); Gordon van Kessel, “Adversary Excesses in the American Criminal Trial” (1992) 67 *Notre Dame Law Review* 403; Mirjan R Damaška, *The Faces of Justice and State Authority: A Comparative Approach to the Legal Process* (Yale UP, 1986).

^20^*R* v *Sean Hoey* [2007] NICC 49.

^21^See John Jackson and Sean Doran, *Judge Without Jury: Diplock Trials in the Adversary System* (OUP, 1995).

^22^The FSS was subsequently, and controversially, closed down to save public money: see House of Commons Science and Technology Committee, *Forensic Science*, Second Report of Session 2013–14, HC 610 (TSO, 2013).

^23^*R* v *Sean Hoey* [2007] NICC 49, [1].

^24^Ibid [51].

^25^“The position so far as [FSNI] is concerned is even more difficult to comprehend as everyone there must have been very well aware of the risks of improper labeling, storage and examination”: Ibid [59].

^26^Ibid [60].

^27^Ibid [60], [61].

^28^Ibid [62].

^29^Ibid [63].

^30^Ibid.

^31^Ibid [64]. Notice that “confident use for evidential purposes” parallels, in my terminology, adequate epistemic warrant for (use in) criminal adjudication.

^32^Ibid.

^33^Brian Caddy, Graham R Taylor and Adrian M T Linacre, *A Review of Low Template DNA Analysis* (2008) [1.8], www.gov.uk/government/uploads/system/uploads/attachment_data/file/117556/Review_of_Low_Template_DNA_1.pdf

^34^Ibid, Executive Summary.

^35^Ibid [3.11].

^36^Ibid [3.15].

^37^Ibid [9.3], [8.1].

^38^Ibid [7.4].

^39^Ibid [7.5].

^40^Forensic Science Regulator, *Response to Professor Brian Caddy's Review of the Science of Low Template DNA Analysis* (FSR, 2008) [4.1.1].

^41^Ibid [4.1.2]. Also now see Peter Gill, June Guiness and Simon Iveson, *The Interpretation of DNA Evidence (Including Low-Template DNA)*, FSR-G-202 (Forensic Science Regulator, 2012); Roberto Puch-Solis, Paul Roberts, Susan Pope and Colin Aitken, *Assessing the Probative Value of DNA Evidence* (Royal Statistical Society, 2012), www.rss.org.uk/statsandlaw.

^42^Forensic Science Regulator, *Response to Professor Brian Caddy's Review of the Science of Low Template DNA Analysis*, [4.1.3].

^43^*R* v *Reed and Reed*; *R* v *Garmson* [2010] 1 Cr App R 23, [2009] EWCA Crim 2698.

^44^Ibid [39].

^45^Ibid [45].

^46^Ibid [49].

^47^Ibid [74].

^48^Ibid.

^49^Ibid.

^50^The leading case remains *R* v *Turner* [1975] 1 QB 834 (CA), elucidating a generic test of “helpfulness”; though this must now be read through the quasi-legislative effect of *Criminal Practice Directions 2015* [2015] EWCA Crim 1567, CPD V Evidence 19A, propounding criteria of admissibility modeled on proposals by the Law Commission. See further, Ian Dennis, “Editorial: Tightening the Law on Expert Evidence” [2015] *Criminal Law Review* 1; Tony Ward, “Expert Evidence and the Law Commission: Implementation without Legislation?” [2013] *Criminal Law Review* 561.

^51^See Paul Roberts, “Expert Evidence and Criminal Trial Procedure” in Gerben Bruinsma and David Weisburd (eds.), *Encyclopedia of Criminology and Criminal Justice* (Springer, 2013) 1480–1494; Paul Roberts and Adrian Zuckerman, *Criminal Evidence* (OUP, 2/e 2010) ch 11.

^52^“[U]nless the admissibility is challenged, the judge will admit that evidence. That is the only pragmatic way in which it is possible to conduct trials”: *R* v *Reed and Reed*; *R* v *Garmson* [2010] 1 Cr App R 23, [2009] EWCA Crim 2698, [113].

^53^For further critical discussion, see Christopher Lawless, *Forensic Science: A Sociological Introduction* (Routledge, 2016), 107–114 and further sources cited therein. As a legal scholar, I am patently unqualified to second-guess Caddy's evaluation of LTDNA profiling techniques, nor do I express any view on the matter. My analysis relates only to English courts' use of the Caddy Review as an authoritative source of information and public endorsement of LTDNA's scientific credentials.

^54^Cf. *R* v *Henderson; R* v *Butler; R* v *Oyediran* [2010] 2 Cr App R 24, [2010] EWCA Crim 1269, discussed in Paul Roberts, “Fue el Bebé Sacudido? Preuba, Pericia y Epistemologia Juridica en el Proceso Penal Inglés” in Carmen Vázquez (ed.), *Estandares de prueba y prueba cientifica* (Marcial Pons, 2013).

^55^On the evolution of DNA profiling techniques, see Michael Lynch, Simon A Cole, Ruth McNally and Kathleen Jordan, *Truth Machine: The Contentious History of DNA Fingerprinting* (Chicago UP, 2008).

^56^Cf. *R* v *McIlkenny* (1991) 93 Cr App R 287 (CA); *R* v *Maguire* (1992) 94 Cr App R 133 (CA); Sir John May, *Inquiry into the Circumstances Surrounding the Convictions Arising Out of the Bomb Attacks on Guildford and Woolwich in 1974*, HC Paper 556 (1990) and HC Paper 296 (1992); Mike Redmayne, “Expert Evidence and Scientific Disagreement” (1997) 30 *UC Davis Law Reviw* 1027; Clive Walker and Russell Stockdale, “Forensic Evidence and Terrorist Trials in the United Kingdom” (1995) 54 *Cambridge Law Journal* 69.

^57^*R* v *Smith (Peter)* [2011] 2 Cr App R 16, [2011] EWCA Crim 1296. See further, Simon A Cole and Andrew Roberts, “Certainty, Individualization and the Subjective Nature of Expert Fingerprint Evidence” [2012] *Criminal Law Review* 824; Jennifer L. Mnookin, “The Validity of Latent Fingerprint Identification: Confessions of a Fingerprinting Moderate” (2008) 7 *Law, Probability and Risk* 127; Robert Epstein, “Fingerprints Meet *Daubert*: The Myth of Fingerprint “Science” is Revealed” (2002) 75 *Southern California Law Review* 605.

^58^*R* v *Reed and Reed*; *R* v *Garmson* [2010] 1 Cr App R 23, [2009] EWCA Crim 2698, [107].

^59^Ibid [103].

^60^See the sources cited at n.41, above.

^61^This was the real issue in *R* v *Adams* [1996] 2 Cr App R 467 (CA); and *R* v *Adams (No 2)* [1998] 1 Cr App R 377 (CA).

^62^*R* v *Broughton* [2010] EWCA Crim 549.

^63^Ibid [31].

^64^*R* v *C* [2010] EWCA Crim 2578, [24], [27].

^65^*R* v *Broughton* [2010] EWCA Crim 549, [37].

^66^Ibid [34].

^67^The present incumbent is Dr Gillian Tully: “Appointment of New Forensic Science Regulator Announced,” Home Office Press Release, 17 July 2014. Her predecessor, in post when *Broughton* was decided, was Andrew Rennison.

^68^*R* v *Broughton* [2010] EWCA Crim 549, [37]. Note that RMPs are not themselves “statistics,” but rather probabilistic extrapolations from allele frequency statistics sampled from reference populations.

^69^Ibid [38].

^70^The Bar Council and Bar Standards Board for barristers; the Law Society and Solicitors Regulation Authority for solicitor-advocates.

^71^*R* v *Broughton* [2010] EWCA Crim 549, [14].

^72^On structural features of “market forensics,” see Christopher J Lawless, “Policing Markets: The Contested Shaping of Neo-Liberal Forensic Science” (2011) 51 *British Journal of Criminology* 671; Paul Roberts, “What Price a Free Market in Forensic Science Services? The Organization and Regulation of Science in the Criminal Process” (1996) 36 *British Journal of Criminology* 37.

^73^Also now a requirement of ECHR Article 6: “According to the Court's settled case law, the existence of impartiality for the purposes of Article 6(1) must be determined according to: (i) a subjective test, where regard must be had to the personal conviction and behavior of a particular judge—that is, whether the judge held any personal prejudice or bias in a given case; and (ii) an objective test, that is to say by ascertaining whether the tribunal itself and, among other aspects, its composition, offered sufficient guarantees to exclude any legitimate doubt in respect of its impartiality.…What is at stake is the confidence which the courts in a democratic society must inspire in the public”: *Volkov* v *Ukraine* (2013) 57 EHRR 1, [104], [106]; *Borgers* v *Belgium* (1993) 15 EHRR 92.

^74^Also see Paul Roberts, Colin Aitken and Graham Jackson, “From Admissibility to Interpretation: New Guidance on Expert Evidence” (2015) 179 *Criminal Law and Justice Weekly* 538 (Part I) and 564 (Part II).

^75^Roberts and Zuckerman, *Criminal Evidence*, ch 15.

^76^See e.g., *R* v *Henderson* [2010] 2 Cr App R 24, [2010] EWCA Crim 1269, [215]–[220]; *R* v *Flynn and St John* [2008] 2 Cr App R 20, [2008] EWCA Crim 970; *R* v *Luttrell* [2004] 2 Cr App R 31, [2004] EWCA Crim 1344, [42], [43]: “The general principle…is that a “special warning” is necessary if experience, research or common sense has indicated that there is a difficulty with a certain type of evidence that requires giving the jury a warning of its dangers and the need for caution, tailored to meet the needs of the case. This will often be the case where jurors may be unaware of the difficulty, or may insufficiently understand it. The strength of the warning and its terms will depend on the nature of the evidence, its reliability or lack of it, and the potential problems it poses.”

^77^Notably, *R* v *Doheny and Adams* [1997] 1 Cr App R 369 (CA). In relation to contested LTDNA profiling evidence, see *R* v *Thomas* [2011] EWCA Crim 1295.

^78^Quoted in *R* v *Broughton* [2010] EWCA Crim 549, [41].

^79^i.e., RMP calculations, (mis)characterized by the trial judge as “the statistical figure that has been given as a match probability”: ibid [43].

^80^Ibid.

^81^Ibid [49].

^82^Ibid [48], [49].

^83^Ibid [49].

^84^*R* v *Sean Hoey* [2007] NICC 49.

^85^See e.g., *R* v *Gordon* [1995] 1 Cr App R 290 (CA). For historical discussion going back to the first trial in which DNA profiling evidence was adduced in 1987, see Paul Roberts, “Forensic Science and Criminal Justice” in Anthea Hucklesby and Azrini Wahidin (eds), *Criminal Justice* (OUP, 2/e 2013); Peter Alldridge, “Recognizing Novel Scientific Techniques: DNA as a Test Case” [1992] *Criminal Law Review* 687.

^86^*R* v *Reed and Reed*; *R* v *Garmson* [2010] 1 Cr App R 23, [2009] EWCA Crim 2698.

^87^*R* v *Broughton* [2010] EWCA Crim 549, [36].

